# Tracking the Progression of Triple Negative Mammary Tumors over Time by Chemometric Analysis of Urinary Volatile Organic Compounds

**DOI:** 10.3390/cancers13061462

**Published:** 2021-03-23

**Authors:** Mark Woollam, Luqi Wang, Paul Grocki, Shengzhi Liu, Amanda P. Siegel, Maitri Kalra, John V. Goodpaster, Hiroki Yokota, Mangilal Agarwal

**Affiliations:** 1 Department of Chemistry and Chemical Biology, Indiana University-Purdue University, Indianapolis, IN 46202, USA; mwoollam@iu.edu (M.W.); pgrocki@iu.edu (P.G.); apsiegel@iupui.edu (A.P.S.); jvgoodpa@iupui.edu (J.V.G.); 2 Integrated Nanosystems Development Institute, Indiana University-Purdue University, Indianapolis, IN 46202, USA; luqiwang@ccmu.edu.cn (L.W.); liu441@iupui.edu (S.L.); hyokota@iupui.edu (H.Y.); 3 Department of Biomedical Engineering, Indiana University-Purdue University, Indianapolis, IN 46202, USA; 4 Hematology and Oncology, Ball Memorial Hospital, Indiana University Health, Muncie, IN 47303, USA; mkalra@IUHealth.org; 5 Biomechanics and Biomaterials Research Center, Indiana University-Purdue University, Indianapolis, IN 46202, USA; 6 Department of Mechanical & Energy Engineering, Indiana University-Purdue University, Indianapolis, IN 46202, USA

**Keywords:** volatile organic compounds (VOCs), gas chromatography (GC), mass spectrometry (MS), headspace solid phase microextraction (HS-SPME), breast cancer biomarkers, principal component analysis (PCA), linear discriminant analysis (LDA), principal component regression (PCR)

## Abstract

**Simple Summary:**

Volatile organic compounds (VOCs) in urine have been shown to be potential biomarkers for breast cancer. However, how urinary VOCs change upon the course of tumor progression has never been studied. The aim of our study was to identify changes in VOC profiles corresponding to mammary tumor (triple negative cells) presence and progression in mice models of induced breast cancer. Urine samples were collected from mice prior to tumor injection and from days 2–19 after. VOC models constructed by linear discriminant analysis had high ability to distinguish tumor-bearing mice from control and determine the week of urine collection after tumor injection. Principal component regression analysis demonstrated that VOCs could predict the number of days since tumor injection. VOCs identified from these analyses correspond to metabolic pathways dysregulated by breast cancer and previous biomarker investigations. It is anticipated that these findings can be translated into human research for early detection of breast cancer recurrence.

**Abstract:**

Previous studies have shown that volatile organic compounds (VOCs) are potential biomarkers of breast cancer. An unanswered question is how urinary VOCs change over time as tumors progress. To explore this, BALB/c mice were injected with 4T1.2 triple negative murine tumor cells in the tibia. This typically causes tumor progression and osteolysis in 1–2 weeks. Samples were collected prior to tumor injection and from days 2–19. Samples were analyzed by headspace solid phase microextraction coupled to gas chromatography–mass spectrometry. Univariate analysis identified VOCs that were biomarkers for breast cancer; some of these varied significantly over time and others did not. Principal component analysis was used to distinguish Cancer (all Weeks) from Control and Cancer Week 1 from Cancer Week 3 with over 90% accuracy. Forward feature selection and linear discriminant analysis identified a unique panel that could identify tumor presence with 94% accuracy and distinguish progression (Cancer Week 1 from Cancer Week 3) with 97% accuracy. Principal component regression analysis also demonstrated that a VOC panel could predict number of days since tumor injection (*R*^2^ = 0.71 and adjusted *R*^2^ = 0.63). VOC biomarkers identified by these analyses were associated with metabolic pathways relevant to breast cancer.

## 1. Introduction

Breast cancer is estimated to comprise 30% of total diagnosed cancer cases for women in 2021: over 280,000 patients will be diagnosed with and over 40,000 patients will die from breast cancer [1]. Accurate and efficient screening/diagnostics is crucial, as the earlier breast cancer is detected, the more efficacious the treatment [2]. Biopsies are used for diagnostic confirmation and pathological grading. Breast cancer staging is based on tumor size and number of lymph nodes affected, but aggressiveness depends on its dynamic rate of change. Breast cancer imaging techniques are used for monitoring tumor progression and morphological responses to treatment [3]. These tools are expensive, unreliable, cause harm through exposure to radiation [4] and can only detect morphological changes six to eight weeks after treatment [5]. There is a growing need for a less invasive and more accurate tool for early detection at the time of diagnosis or for cancer recurrence. An accurate and noninvasive assay to diagnose and monitor tumor progression could aid in patient decision making after diagnosis and possibly during treatment.

Previous studies have demonstrated canine’s ability to detect the presence of prostate [6], lung [7], breast [8,9] and ovarian [10] cancer in biosamples with high accuracy [11,12]. Canines noninvasively detect volatile metabolites generated by the disease condition, allowing them to accurately detect cancer by scent. Based on canine results, groups have used headspace solid phase microextraction (HS-SPME) or other extraction techniques coupled with gas chromatography-mass spectrometry (GC-MS) to conduct untargeted analyses of volatile organic compounds (VOCs). Cancer dysregulates metabolic pathways to enable tumor growth [13]. The biological rationale for exploiting VOCs is they are by- or end products of these dysregulated pathways [14]. Furthermore, VOCs can be noninvasively sampled and detected in human biofluids including sweat, saliva, blood, breath and urine. Groups have previously identified VOC biomarkers for lung [15], prostate [16,17,18,19] and breast cancer [20], even classifying unique cancers from each other [21,22].

There has been an interest in using VOCs and other types of molecular biomarkers [23,24,25] in urine or breath for breast cancer diagnostics. One group analyzing alveolar breath by GC-MS found ten VOCs, that could distinguish breast cancer in patients with sensitivity = 75.3% and specificity = 84.8% [20]. Another group also detected a unique biosignature of six VOCs for breast cancer in human urine via unsupervised multivariate statistical analysis [26]. That group subsequently implemented a central composite design to optimize method parameters and used them to identify ten additional breast cancer volatile biomarkers that had accuracy of >90% [27]. The present study analyzes urinary VOCs in mice with breast cancer using GC-MS not simply to identify VOCs of breast cancer, but to learn how VOCs change in different conditions associated with breast cancer. For example, we previously analyzed changing patterns in VOCs caused by tumor location [28] or effect of treatments [29,30,31]. Interestingly, our murine research has previously reported almost half the VOCs tentatively identified by a different research group analyzing human breath for VOCs of breast cancer [20] and for the sixteen VOCs reported in the *Silva* papers, which incubated under different conditions, about half if including isomers and other highly similar molecules. Additionally, urinary VOC biomarkers for breast cancer and tumor progression may be useful in conjunction with circulating biomarkers of breast cancer progression. For an example, *Ibrahim* et al. found the receptor activator of nuclear factor-κB ligand (RANKL) to be a potential circulating biomarker for bone metastasis [32]. Correlating the urinary biomarkers from this study to RANKL and other circulating biomarkers which may be identified would help validate the candidates identified in this study. Additionally, circulating tumor and urinary VOCs can be coupled, which could also potentially improve classification accuracies and acceptance of alternative assays for breast cancer diagnosis, prognosis and monitoring the efficacy of treatment.

All of our previous studies analyzed samples from mice collected at the same time point (three weeks after tumor injection); none analyzed VOCs at intermediate points of time. There are many murine models of cancer progression, but in the current study, BALB/c mice were injected with 4T1.2 cells into the tibia. Tumors injected in this way progress and typically produce osteolytic lesions in 1–2 weeks [33]. A previous study by some of the authors, using such a model, found decreases in bone stiffness which would be indicative of osteolytic lesions after one week and before two weeks [34]. Detailed and additional biological information regarding this cohort of mice has been previously published [30]. For the current study, urine samples were collected from day 2–day 19 and grouped by number of weeks after injection. Analyses included comparisons between samples by week collected and regression analysis of samples by day collected to observe trends. Analysis of VOCs over time may help identify differences in VOC patterns to determine which biomarkers are better predictors of late-stage cancers and which are equally effective at identifying early-stage cancers.

## 2. Materials and Methods

### 2.1. Materials and Instrumentation

Female BALB/c mice (6 weeks old) were purchased from Harlan Laboratories, Indianapolis, IN, USA. 4T1.2 tumor cells were acquired from Dr. R. Anderson at the Peter MacCallum Cancer Institute (Melbourne, Victoria, Australia). Glass Pasteur pipettes were used for urine collection and purchased from Thermo Scientific (Waltham, MA, USA). 10 mL headspace vials were purchased from Thermo Scientific. Guanidine Hydrochloride (GHCl) (pH = 8.5) was purchased from Sigma Aldrich (St. Louis, MO, USA) and was used as a major urinary protein (MUP) denaturing agent. Two-centimeter polydimethylsiloxane/carboxen/divinylbenzene (PDMS/CAR/DVB) SPME fibers (Supelco; Bellefonte, PA, USA) were employed to concentrate and extract VOCs. A 7890A GC system coupled to an Agilent (Santa Clara, CA, USA) 7200 Accurate-Mass Quadrupole time-of-flight (QTOF) MS system with a PAL autosampling system (CTC Analytics; Raleigh, NC, USA) was used to analyze VOCs. An Agilent Ultra Inert HP-5ms, GC column of 30 m in length, 250 μm internal diameter and 0.25 μm film thickness was utilized. MATLAB (R2020a; Natick, MA, USA) and Origin (Northampton, MA, USA) were used in generating figures for chemometric analyses.

### 2.2. Tumor Injection and Urine Collection

A total of 20 mice were injected with 4T1.2 cells (triple negative mammary tumors) into the right tibia [30]. Prior to tumor injection, urine was collected from the mice to serve as the Control group. Urine was collected from the 20 mice the day following tumor injection and over the course of three weeks. It is important to note that not all mice provided urine samples at each time point. All experimental procedures followed the Guiding Principles in the Care and Use of Animals supported by the American Physiological Society and were approved by the Indiana University Animal Care and Use Committee (protocol code: SC292R; date of approval: 30 May 2019). Mice were kept in cages at ambient temperature and fed the same diet (mouse-chow ad libitum). Mice were transferred to a cage where the floor was covered in parafilm during urine collection. Urine was collected over dry ice using glass Pasteur pipettes into glass centrifuge tubes and centrifuged at 3000 RPM. A total of 50 μL was transferred to a 10 mL headspace vial and stored in a −80 °C freezer.

### 2.3. HS-SPME and GC-MS QTOF Analysis

Urinary VOCs were detected through headspace analysis utilizing a SPME fiber and GC-MS QTOF. The SPME fiber was conditioned before the first sample each day and between each run. As the mice gave limited amounts of urine, only one aliquot was analyzed per mouse. GHCl was added in a 1:1 volumetric ratio one hour prior to GC analysis to denature the MUPs in mouse urine that bind VOCs [35]. Next, the sample was agitated at 250 rpm and heated to 60 °C for 30 min. Then, the SPME fiber was inserted into the vial for an additional 30 min (same agitation rate and temperature). The fiber was injected into the GC inlet at 250 °C for two minutes to thermally desorb the VOCs. The chromatographic protocol involved maintaining the oven temperature at 40 °C for two minutes followed by a ramp to 100 °C at a rate of 8 °C/min, a 15 °C/min ramp to 120 °C, an 8 °C/min to 180 °C, a 15 °C/min to 200 °C and finally an 8 °C/min ramp to 260 °C. An external reference standard was run each day to verify instrument reproducibility.

### 2.4. Data Treatment and Chemometric Analyses

Deconvolution and spectral alignment of chromatographic peaks based on similarities in mass-to-charge ratio (*m/z*) and retention time were performed in MassHunter Profinder. Features identified as silanes/siloxanes (products of SPME degradation) were removed. VOCs that did not appear in at least 50% of either Control or Cancer Weeks 1–3 samples were also excluded. To normalize the data, MS Total Useful Signal (MSTUS) was calculated and applied to remove unwanted non-biological intraclass variation [36]. Finally, MSTUS values were autoscaled (*z*-scored) to obtain a matrix with similar signal range. Univariate statistical analysis was implemented (two-tailed Student’s *T*-test) on the Control group against tumor-bearing mice with urine collected on days 2, 5 and 6 (Week 1), urine collected on days 8, 9, 12 and 13 (Week 2) and finally, urine collected on days 16, 17 and 19 (Week 3). These analyses were implemented to identify VOCs differentially expressed (*p*-value < 0.05) between Cancer Weeks 1–3 and Control as well as between Cancer Week 1 and Cancer Week 3. *p*-values were adjusted utilizing the Benjamini–Hochberg procedure [37] to account for false discovery rates (FDR). Hierarchical heatmaps were generated for VOCs statistically significant by *p*-value < 0.05 for Control against Weeks 1–3 and Week 1 against Week 3 to visualize changes in VOC concentration induced by cancer injection and progression.

Principal component analysis (PCA) was performed using all VOCs with *p*-value < 0.05 between the Control group and all Cancer groups. PCA was also implemented on a smaller group of VOCs with the lowest *p*-value (for Cancer/Control and Cancer Week 1/Week 3) to separate Control, Cancer Week 1 and Cancer Week 3. The matrix of VOCs was then subject to supervised linear discriminant analysis coupled with forward feature selection (iterative LDA, iLDA) [38] to build predictive classification models. PCA and iLDA were performed independently of each other in parallel. iLDA was used to develop VOC panels separating Control vs. Cancer, Cancer Week 1 vs. Cancer Week 3 and lastly, Cancer Week 1 vs. Cancer Week 3 vs. Control. Leave one out cross validation (LOOCV) and fivefold cross validation (partitioned 1000 times, median value utilized) were performed to determine if the models were overfit [39]. Receiver operator characteristic (ROC) curves for each model were built to visualize classification accuracies. If the area under the curve (AUC) of the ROC differed more than 0.10 between the training and cross validation data sets, the model was deemed overfit.

### 2.5. Regression Analyses

To further investigate the ability of individual VOCs to monitor mammary tumor progression, linear regression analysis was undertaken for VOCs with *p*-value < 0.05 (Cancer Week 1 vs. Cancer Week 3). Here, Cancer samples were analyzed by day of urine collection after tumor injection. Principal component regression (PCR) analysis was also implemented to identify if a panel of VOCs can track mammary tumor progression by days after injection. PCR proceeds by running PCA on the table of the explanatory (input) variables. Then, an Ordinary Least Squares regression is completed on a group of principal components selected by the user. Finally, PCR computes the parameters of the model that correspond to the explanatory (input) variables. The number of principal components utilized was varied and tested to ensure the production of a stable model. The first iteration of analysis was followed by dimension reduction to only include VOCs that significantly contributed toward the PCR model (*p*-value < 0.05). Analysis of variance (ANOVA) was implemented to determine if there was a significant linear correlation between the independent and the dependent variables. Determination coefficients (*R*^2^ value), regression coefficients and standard errors were used to assess the degree of correlation.

### 2.6. VOC Identification and Metabolic Pathway Analysis

After data screening and analysis, volatiles were identified using MassHunter Profinder and MassHunter Unknowns Analysis with the NIST17 library. The data set produced through Profinder contained average retention times, retention time span and the mass spectra of the VOCs. Utilizing these quantifiers, VOCs in Profinder were found in Unknowns Analysis. Features in Unknowns Analysis were assigned a match factor from the NIST17 library; VOCs identified with a match factor greater than 70 and an appropriate experimental non-polar retention index (NPRI) value were deemed tentatively identified. Experimental NPRI was determined using an instrument-specific calibration curve [28,29]. The Human Metabolome Database [40] and Kyoto Encyclopedia of Genes and Genomes Pathways [41] were used to aid in interpreting the relevance of VOCs in the context of cancer metabolism.

## 3. Results

### 3.1. Urine Collection, Spectral Alignment and Data Normalization

**Figure 1:**
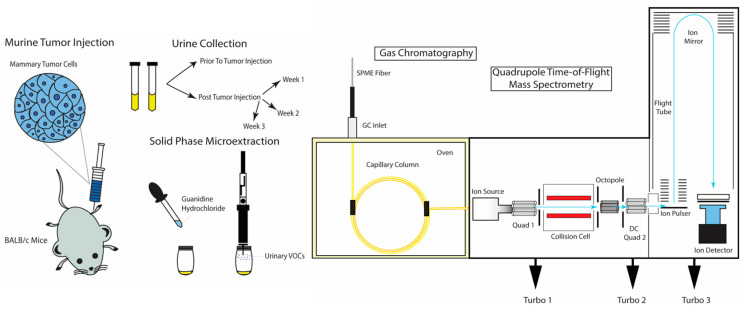
Illustration of murine tumor injection, mouse urine sample collection, sample treatment and analysis via solid phase microextraction coupled to gas chromatography-quadrupole time-of-flight mass spectrometry to identify volatile organic compound (VOC) biomarkers for breast cancer and tumor progression.

An illustration of the experimental procedure that was implemented to identify VOC biomarkers of mammary tumor progression in mouse urine can be visualized in Figure 1. A total of 65 urine samples were collected, aliquoted and analyzed from four different sample classes over the course of three weeks (Control (20), Cancer Week 1 (12), Cancer Week 2 (15) and Cancer Week 3 (18)). Spectral alignment of sample chromatograms generated a matrix of 250 VOCs which were subject to chemometric analyses after removing silanes/siloxanes and volatiles not detected in at least half of either Control or Cancer samples classes.

### 3.2. Univariate Statistical Analysis

**Figure 2:**
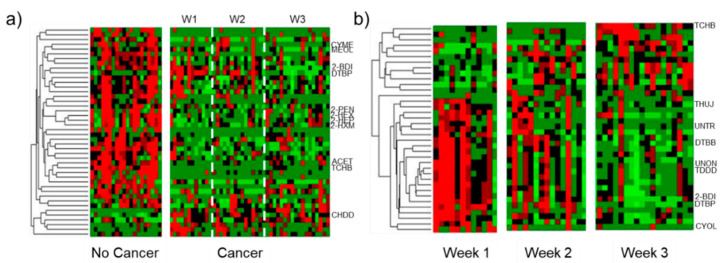
Hierarchical heatmaps for the VOCs identified with *p*-value < 0.05 in the (**a**) Cancer Weeks 1–3 vs. Control and (**b**) Cancer Week 1 vs. Cancer Week 3 comparisons. These plots show an abundant number of VOCs differentially expressed due to the presence of cancer and tumor progression. Full names of VOCs used for further analyses (but here abbreviated) are enumerated in the text and all VOCs shown in the heatmap and associated *p* values are listed in Table S1.

After normalization, Student’s *T*-test was performed between Control and All Cancer classes and identified 44 out of 250 VOCs with *p*-value < 0.05. Additionally, the *T*-test found 37 VOCs with *p*-value < 0.05 when applied between the Cancer Week 1 and Week 3 sample classes. These VOCs are identified and listed in Table S1 with their corresponding name, retention time and *p*-values. After adjusting *p*-values for FDR, 18 VOCs with *p*-value < 0.05 were found between Control and Cancer (italicized in Table S1) and 3 VOCs were identified with *p*-value < 0.05 between Week 1 and Week 3 (underlined in Table S1). Of the 44 VOCs identified with *p*-value < 0.05 between Control and Weeks 1–3, 37 features were downregulated and 7 upregulated. With regards to the 37 significant VOCs between Cancer Week 1 and Week 3, 24 were downregulated and 13 upregulated. Downregulated features were more significant than upregulated ones for both comparisons. Hierarchical heatmaps were generated using statistically significant VOCs between each comparison and are shown in Figure 2a,b. The heatmap corresponding to Control vs. Weeks 1–3, shows low intraclass variation and high interclass variation (Figure 2a). Figure 2b shows many VOCs are downregulated in Week 3, indicating the concentration of these VOCs decreases as cancer progresses. The heatmap displays high intraclass variation for VOCs with *p* < 0.05 detected in Week 2, indicating some mice progressed faster than others, or possibly some mice had undergone tumor-induced osteolysis and some had not yet [30], but no imaging or invasive studies were undertaken during intermediate time points, so this is not confirmed. In Figure 2, abbreviations for VOCs that show high statistical significance and are utilized for further analyses are indicated.

### 3.3. Multivariate Classification Analyses

**Figure 3:**
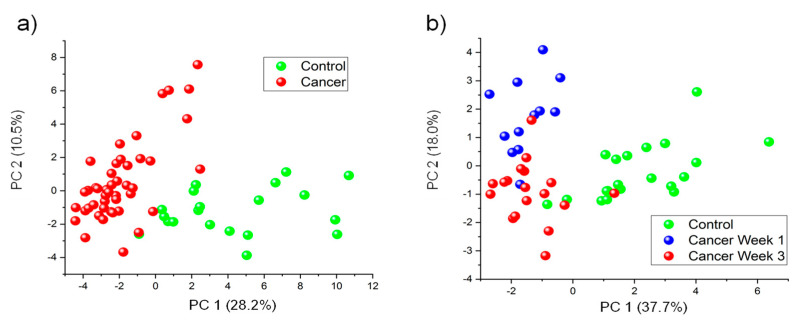
(**a**) PCA using all VOCs with *p* < 0.05 between all Cancer samples and Control samples (44 VOCs). This panel can distinguish Cancer Weeks 1−3 from Control with high accuracy. (**b**) PCA using 10 VOCs separating Cancer Week 1, Cancer Week 3 and Control samples. This smaller panel of VOCs can separate these classes with good accuracy.

PCA was utilized to visualize global patterns in the data. PCA using all 44 VOCs with *p*-value < 0.05 between Cancer Weeks 1–3 and Control can be observed in Figure 3a. Along the first two principal components, all Cancer samples were separated from Control samples with 98% sensitivity and 95% specificity. A smaller panel of 10 VOCs with low *p*-values for both tumor presence and progression were selected using an ad hoc approach and PCA was run using this smaller set (Figure 3b). For this analysis, Cancer Week 2 was excluded as it is intermediary and the goal was to observe significant differences at the two endpoints. Cancer Week 1 and Week 3 samples were separated from Control samples with sensitivity = 97% and specificity = 90%. Principal component 1 demonstrates sample separation between Cancer and Control samples and Principal component 2 strongly contributed toward sample separation between Cancer Week 1 and Cancer Week 3. VOCs used in Figure 3b are labeled in Table S1 with an asterisk (*).

**Figure 4:**
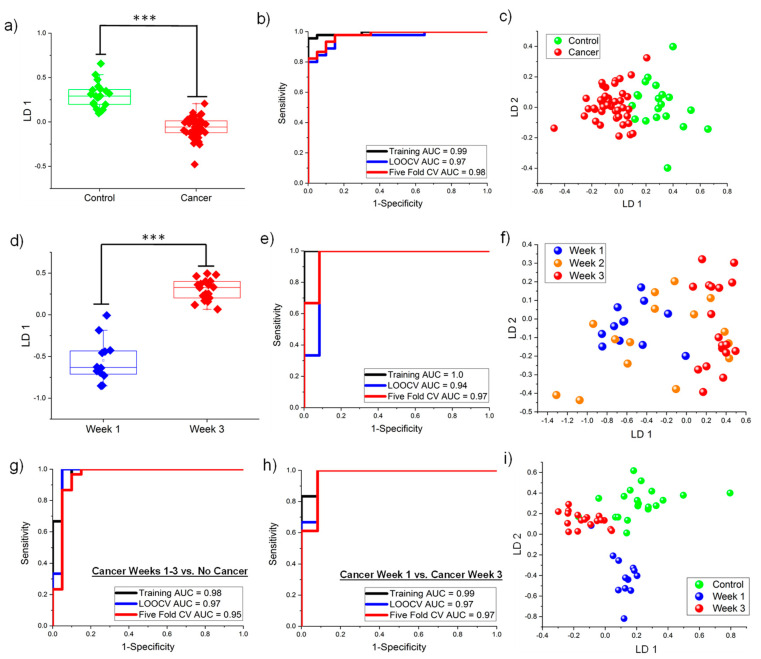
(**a**–**c**) First LDA Model distinguishes all Cancer (Weeks 1–3) from Control with high accuracy using five VOCs showing results in (**a**) one dimension, (**b**) the receiver operator characteristic (ROC), and (**c**) First LDA model in two dimensions. (**d**–**f**) Second LDA Model distinguishes Cancer Week 1 from Cancer Week 3 with high accuracy showing results using five VOCs in (**d**) one dimension, (**e**) the ROC for Week 1 vs. Week 3, and (**f**) Second LDA model in two dimensions. Week 2 samples shown in (**f**) not included in (**d**) LD1 or (**e**) ROC analysis. (**g**–**i**) Third LDA Model separates Cancer Week 1, Cancer Week 3 and Control with high accuracy. The ROC curves are shown for (**g**) Cancer Weeks 1 and 3 vs. Control and (**h**) the Cancer Week 1 vs. Week 3. (**i**) The third LDA model in two dimensions. *** *p* < 0.001.

Even though PCA produced a reasonable separation, this required a relatively large number of VOCs. To build a predictive classification model and decrease the number of VOCs used, iLDA was used to distinguish Cancer Weeks 1–3 from Control samples. Knowledge-based feature selection was implemented by limiting the analysis to ketones, aromatics and terpenes as these functional groups have been previously reported by our team to be potential biomarkers [28,29]. A panel of five VOCs (cymene (CYME), acetone (ACET), 2-heptanone (2-HEP), 2,5-cyclohexadiene-1,4-dione, 2,6-bis(1,1-dimethylethyl)- (CHDD) and 2-hexanone, 5-methyl (2-HXM)) could classify tumor presence (Cancer Weeks 1–3 from Control) with an AUC equal to 0.99 in the training set (sensitivity = 98% and specificity = 95%) (the First LDA Model). The one-dimensional LDA plot can be observed in Figure 4a and it is clear the first linear discriminant accounted for the significant differences between the two sample classes. Data perturbation techniques were implemented to test the robustness of the classification models [39,42]. LOOCV (AUC = 0.97) and fivefold cross validation (AUC = 0.98) showed values similar to the training data, demonstrating the model was not overfit. The respective ROC curves can be seen in Figure 4b and the two-dimensional LDA plot is illustrated in Figure 4c.

iLDA was also implemented on a subset of ketones, terpenes and aromatics to model changes in murine VOCs between Cancer Week 1 and Cancer Week 3. iLDA identified a different biosignature of five VOCs (p-Cymen-8-ol (CYOL), 1,3,5-Undecatriene (UNTR), 8,8,9-Trimethyl-deca-3,5-diene-2,7-dione (TDDD), 2,4-Di-tert-butylphenol (DTBP) and 2-Butanone, 3,3-dimethyl- (2-BDI)) that classified Cancer Week 1 from Cancer Week 3 with 100% accuracy in the training data set (AUC = 1.0) (The Second LDA Model). Use of the first linear discriminant led to a perfect separation and one-dimensional LDA box/whisker plots can be observed in Figure 4d. LOOCV (AUC = 0.94) and fivefold cross validation (AUC = 0.97) were implemented and showed the model was not overfit (ROC in Figure 4e). Cancer Week 2 samples were tested using this model and the two-dimensional LDA plot can be seen in Figure 4f, which shows that some of the samples clustered in between Cancer Week 1 and Week 3, some clustered with Cancer Week 1 and some clustered with Cancer Week 3. This mirrors the results from the hierarchical heatmap in Figure 2b. Week 2 samples were not included in the statistical analysis comparing Weeks 1 and 3, shown in Figure 4d,e.

Next, the team undertook iLDA to identify a single panel capable of distinguishing all three sample classes of interest (Control, Cancer Week 1 and Cancer Week 3). iLDA was undertaken and applied on all VOCs that were differentially expressed for all sample comparisons (including VOCs only significant in Week 3 of Cancer). LDA utilizing five compounds (damascenone, 1,3,5-trichlorobenzene (TCHB), linalool, DTBP and 2-hexanone (2-HEX)) led to accurate classification of all three sample classes (the Third, LDA Model). Linalool is a linear monoterpenoid and damascenone an isoprenoid lipid that are not listed in Table S1 because although statistically significant for Control/Week 3 classification, they were not univariately significant for either All Cancer/Control or the Cancer Week 1/Week 3 comparison. Using this third LDA model, Cancer samples were distinguished from Control samples with an AUC equal to 0.98, sensitivity = 100% and specificity = 95% (LOOCV AUC = 0.97 and fivefold cross validation = 0.95). Alternatively, Cancer Week 1 was classified from Week 3 with AUC = 0.99, sensitivity = 100% and specificity = 92% (LOOCV AUC = 0.97 and fivefold cross validation AUC = 0.97). The ROC curves can be seen in Figure 4g,h, while the two-dimensional LDA plot can be observed in Figure 4i. Urine samples collected from the Week 2 cohort were tested using this classification model and the LDA plot is shown in Figure S1. Week 2 samples are 100% distinguished from Control samples and again cluster in between Cancer Week 1 and Cancer Week 3. However, many of the mouse urine samples were classified as either Cancer Week 1 or Cancer Week 3, showing that some of the mice in Cancer Week 2 had tumors that may have progressed faster.

### 3.4. Linear and Principal Component Regression Analysis

**Figure 5:**
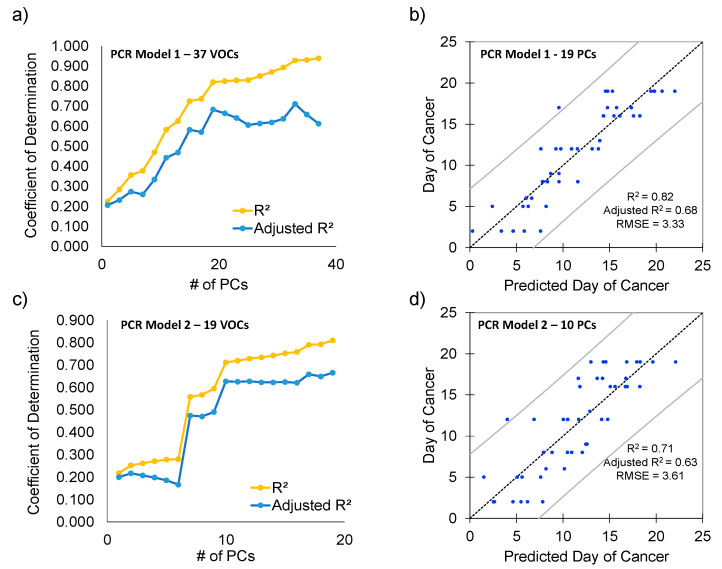
Principal component regression (PCR) analysis using models starting with 37 and 19 VOCs, respectively, identify leaner models in an iterative fashion. (**a**) Coefficient of determination plotted against the number of principal components utilized for the principal component regression (PCR) analysis using 37 VOCs with *p*-value < 0.05 (Cancer Week 1 vs. Cancer Week 3). (**b**) PCR analysis using the first 19 principal components with calculated *R*^2^ equal to 0.82 and adjusted *R*^2^ equal to 0.68. (**c**) Coefficient of determination plotted against the number of principal components utilized for the PCR analysis using 19 VOCs significantly contributing toward the linear correlation. (**d**) PCR analysis using the first 10 principal components results in a more stable model which could predict the number of days after tumor injection with *R*^2^ equal to 0.71 and adjusted *R*^2^ equal to 0.63.

Linear regression analysis was undertaken on individual VOCs (37 with *p*-value by Student’s *t*-test < 0.05 between Cancer Week 1 and Week 3) to look for significant trends with respect to the day on which urine was collected after tumor injection. Determination coefficients (*R*^2^ value), regression coefficients and standard errors for all 37 individual VOCs can be observed in Supplementary Table S2. ANOVA determined that 23 out of the 37 VOCs had statistically significant trends by linear regression (*p*-value < 0.05). Of the 23 VOCs, only 5 VOCs had a positive regression coefficient while the other 18 features had negative regression coefficients. Even though statistically significant correlations were observed, none of the VOCs had an adjusted *R*^2^ greater than 0.35. Next, PCR was implemented on the same 37 VOCs. After employing PCR on all 37 principal components, a relatively good fit was obtained (*R*^2^ = 0.94), but the adjusted *R*^2^ value was much lower (0.61), indicating too many variables were being utilized. A scatter plot showing the *R*^2^ and adjusted *R*^2^ value as a function of the number of principal components utilized for this model can be seen in Figure 5a, which indicates that analyzing more than 19 principal components will result in an overfit model. PCR using the first 19 components resulted in a linear correlation with *R*^2^ = 0.82, adjusted *R*^2^ = 0.68 and root mean square error (RMSE) = 3.3 (Figure 5b). The 95% confidence interval for the linear regression model is additionally illustrated in Figure 5b. The standardized coefficients for all 37 VOCs can be observed in Table S2. To increase the stability of the model, PCR was implemented using only the 19 VOCs (principal component loadings) statistically significantly contributing toward the first iteration of regression analysis (Figure 5b). Again, when plotting the *R*^2^ and adjusted *R*^2^ value as a function of the number of principal components (Figure 5c), utilizing more than 10 principal components results in an overfit model. The first 10 principal components resulted in a linear correlation with *R*^2^ = 0.71, adjusted *R*^2^ = 0.63 and RMSE = 3.6 (Figure 5d). The 95% confidence interval for this PCR model is can also be observed in Figure 5d. The standardized coefficients for all 19 VOCs for this model can also be observed in Table S2.

## 4. Discussion

The heatmaps (Figure 2) are consistent with previous studies in murine models of breast cancer [28,29]; more VOCs are downregulated by cancer. However, previous studies found roughly twice as many volatile terpenes and terpenoids among the cancer biomarkers as ketones [28]. In the current study, ketones were observed to be more consistently dysregulated by mammary tumors, regardless of when the urine was collected (Week 1, Week 2 or Week 3). PCA and iLDA had the ability to classify all Cancer samples with over 95% classification accuracy (Figure 3a and Figure 4a–c) using different panels of VOCs. Multivariate analyses could even distinguish the three sample classes of interest (Cancer Week 1, Cancer Week 3 and Control) with over 90% accuracy (Figure 3b and Figure 4g–i) based on different VOCs. These models show the ability of VOCs to classify any type of Cancer and distinguish progression by week after tumor injection with outstanding accuracy.

PCR of all 37 VOCs with *p*-value < 0.05 and utilizing the first 19 principal components resulted in a stable linear model (Figure 5a,b). Upon limiting principal component regression to 19 VOCs, a model utilizing the first ten principal components resulted in a model with greater stability (Figure 5c,d). Both of the principal component regression models presented have a higher degree of linear correlation relative to any individual VOC (Table S2). The 19 VOCs identified from principal component regression as useful for tracking tumor progression by day correspond to the results from multivariate classification of Cancer by week. For example, five of the ten VOCs (DTBP, 2-BDI, CYOL, THUJ and TCHB) that contribute toward the separation of the Control, Cancer Week 1 and Week 3 in PCA (Figure 3b) were identified by PCR. On the other hand, four of the five VOCs identified by LDA (CYOL, TDDD, 2-BDI and DTBP) to separate Cancer Week 1 from Cancer Week 3 (Figure 4d–f) were found to be significant by PCR. Two of the VOCs identified by LDA to classify Control, Cancer Week 1 and Week 3 (TCHB and DTBP, shown in Figure 4g–i) contributed toward PCR.

Ketones were the most frequent functional group detected as significantly dysregulated as shown in Table S1. This is consistent with our previous analysis which showed ketones were depleted in tumor-bearing mice [28]. Ketones have been previously reported by *Silva* et al. to be potential markers for breast cancer as they are products of lipid peroxidation [43]. Ketones and other carbonyls have been reported to be markers for prostate cancer [16,18], lung cancer [44,45] and diabetes [46]. Another study by the authors showed two ketones reported in this study, 2-HEP and 2-PEN, were enriched in urine samples collected from mice receiving an antitumoral treatment [47] (treatment was bone loading, a simulated form of exercise). Furthermore, in vitro analyses showed upon treatment with 2-HEP and 2-PEN, hypothalamic neuronal cells had reduced tumor cell viability accompanied with elevated levels of aralkylamine N-acetyltransferase (AANAT) and tyrosine hydrogenase (TH). AANAT and TH are rate-limiting enzymes that produce melatonin and dopamine, which have been shown to have a role in tumor suppression [47]. These studies show the potential antitumor capability of ketones, which is intriguing as they were downregulated by mammary tumors in this study.

Volatile terpenes/terpenoids (VTs) were found to be depleted in Cancer samples (specifically in Week 3). VTs were previously identified to be potential markers of mammary tumors in mice [28,29] and are synthesized in vivo by the mevalonate (MVA) pathway. The MVA pathway has not only been shown to be dysregulated by cancer, but also to play a significant role in tumor growth and transformation [48,49]. The change in VT profiles may be due to osteolytic lesions likely forming by Week 3. Down-regulation of the MVA pathway through the use of a class of HMG-CoA reductase inhibitors known as statins is known to slow osteolysis in breast cancer models [30,50,51]. Cholesterol, an end product downstream of the MVA pathway, has also been reported to play a role in tumor growth [52]. The current authors previously have shown a correlation between the dysregulation of urinary VTs and the upregulation of cholesterol in mice [30]. This is important, as both VTs and cholesterol are products of the MVA pathway. Lastly, there is a link between VTs as markers of cancer and potential treatments, because they have demonstrated inhibitory effects against cancer [53,54,55].

2,4-Di-tert-butylphenol (DTBP) and 3,5-di-tert-Butyl-4-hydroxybenzaldehyde (DTBB), which are classified as phenolic antioxidizing agents [40], are two aromatic VOCs identified in the study. DTBB does not show any significant differences between Control samples and Cancer Week 1 but becomes increasingly depleted by Week 3. On the other hand, DTBP is significantly enriched in Cancer Week 1 relative to Control samples but becomes progressively depleted by Cancer Week 3. These aromatic VOCs are potentially of interest because natural phenolic antioxidant agents are secondary metabolites and their ability to serve as an anticancer therapy has been previously analyzed. Studies have shown that several groups of phenolic antioxidants inhibit the growth and proliferation of tumor cells, but the mechanism of action has not been entirely elucidated [56]. Given the previously demonstrated antitumor capability, it is unsurprising to see their depletion induced by tumor progression and/or the formation of osteolytic lesions.

Taken together, these results show that VOCs can not only classify tumor-bearing mice, but also accurately track progression. Limitations of this study include the relatively small number of samples analyzed. Additionally, the metabolic variation of all mice was relatively controlled: BALB/c mice were the same age, given the same tumors (triple negative 4T1.2 cells), kept in the same environment and fed the same diet. Women have varied breast cancer tumors, larger variability in age, exercise, diet and other lifestyle factors. However, it is evident most of the VOCs in the murine study do not represent the tumors themselves, but the metabolic response to tumors; a human metabolic response to tumors would be expected to also present significant similarities despite differences in tumor type and the other variations noted above. In the future, it may be fruitful to combine VOC analysis with other predictors of tumor progression such as the identified circulating biomarker for bone metastasis [32] and others which have not yet been identified.

## 5. Conclusions

It is important, when translating results from murine models to human studies, to recollect that murine samples may represent late stages in cancer tumor progression. This study found tumor injection to the tibia led to many VOCs being dysregulated as early as the first week after injection. Some VOCs remained relatively constant over the course of the study, while others were insignificant early in the study but were dysregulated later in the study. This is hypothesized to be because of cancer progression and/or the formation of osteolytic lesions induced by mammary tumor injection/progression. It is hoped these findings can be translated into human research for early detection of breast cancer recurrence as 20–30% of patients with early breast cancer will experience relapse with distant metastatic disease and bone metastases being the most common presentation at the time of recurrence [2]. Further human research can be focused on finding if the urinary VOCs can be detected before the radiographic appearance of lesions on the bone scans or PET scans, or exploring if there is any correlation between the levels of VOCs and the extent of tumor burden.

## Data Availability

The data presented in this study, are available on request from the corresponding author.

## Supplementary Materials

The following are available online at https://www.mdpi.com/2072-6694/13/6/1462/s1, Table S1: List of VOCs with p-value < 0.05 between Cancer Weeks 1–3 vs. Control (tumor presence) or Cancer Week 1 vs. Cancer Week 3 (tumor progression) with corresponding abbreviations, retention times and p-values for both comparisons (NS = no statistical significance, upward facing arrows show upregulation in all Cancer samples or in Cancer Week 3 while downward facing arrows signify downregulation, * denotes VOCs used for PCA in Figure 3b, underline denotes *p*-value < 0.05 between Cancer Week 1 and Week 3 after FDR adjustment, italics denotes *p*-value < 0.05 between Cancer and No Cancer after FDR adjustment). Table S2. Regression analysis results of individual 37 VOCs identified as significantly different (Cancer Week 1 and Week 3), standardized regression coefficients for the same 37 VOCs analyzed by PCR in Figure 5b and standardized regression coefficients for the 19 VOCs analyzed by PCR in Figure 5d. Figure S1: 2D LDA plot with Cancer Week 2 samples tested utilizing the LDA model initially built to discriminate Control, Cancer Week 1 and Cancer Week 3.
