# Supplementary material for: Tracking the Progression of Triple Negative Mammary Tumors over Time by Chemometric Analysis of Urinary Volatile Organic Compounds
Source: Cancers (Basel). 2021 Mar 23;13(6):1462. doi: 10.3390/cancers13061462 (PMC8004946; doi:10.3390/cancers13061462)
Supplement: Supplementary file 1 [file cancers-13-01462-s001.pdf]

Supplementary Information

Tracking the Progression of Triple Negative Mammary Tumors Over Time by Chemometric Analysis of Urinary Volatile Organic Compounds

Mark Woollam[1, 2], Luqi Wang [2,3], Paul Grocki[1,2], Shengzhi Liu[2,3], Amanda P. Siegel[1,2], Maitri Kalra[4], John V. Goodpaster[1], Hiroki Yokota[2,3,5] and Mangilal Agarwal[1,2,6*]

[1] Department of Chemistry and Chemical Biology, Indiana University-Purdue University, Indianapolis, IN 46202, USA
[2] Integrated Nanosystems Development Institute, Indiana University-Purdue University, Indianapolis, IN 46202, USA
[3] Department of Biomedical Engineering, Indiana University-Purdue University, Indianapolis, IN 46202, USA
[4] Indiana Health Ball Memorial Hospital, Muncie, IN 47303, USA
[5] Biomechanics and Biomaterials Research Center, Indianapolis, IN 46202, USA
[6] Department of Mechanical & Energy Engineering, Indiana University-Purdue University, Indianapolis, IN 46202, USA
[*] Correspondence: agarwal@iupui.edu

Table of Contents

Table S1: List of VOCs with p-value < 0.05 between Cancer Weeks 1-3 vs. Control (tumor presence) or Cancer Week 1 vs. Cancer Week 3 (tumor progression) with corresponding abbreviations, retention times and *p*-values for both comparisons (NS=no statistical significance, upward facing arrows show upregulation in all Cancer samples or in Cancer Week 3 while downward facing arrows signify downregulation, * denotes VOCs used for PCA in Figure 3(b), underline denotes *p*-value < 0.05 between Cancer Week 1 and Week 3 after FDR adjustment, *italics* denotes *p*-value < 0.05 between Cancer and No Cancer after FDR adjustment).

Table S2: Regression analysis results of individual 37 VOCs identified as significantly different (Cancer Week 1 and Week 3), standardized regression coefficients for the same 37 VOCs analyzed by PCR in Figure 5(b) and standardized regression coefficients for the 19 VOCs analyzed by PCR in Figure 5(d).

Figure S1: 2D LDA plot with Cancer Week 2 samples tested utilizing the LDA model initially built to discriminate Control, Cancer Week 1, and Cancer Week 3.

Table S1. List of VOCs with p-value < 0.05 between Cancer Weeks 1-3 vs. Control (tumor presence) or Cancer Week 1 vs. Cancer Week 3 (tumor progression) with corresponding abbreviations, retention times and *p*-values for both comparisons (NS=no statistical significance, upward facing arrows show upregulation in all Cancer samples or in Cancer Week 3 while downward facing arrows signify downregulation, * denotes VOCs used for PCA in Figure 3(b), underline denotes *p*-value < 0.05 between Cancer Week 1 and Week 3 after FDR adjustment, *italics* denotes *p*-value < 0.05 between Cancer and No Cancer after FDR adjustment).

**Name**	**Abbr.**	**Retention Time**	**Cancer/No Cancer *p*-value**	**Week 1/Week 3 *p*-value**
*2H-Pyran-2,3-diol, tetrahydro-, diacetate, cis-	PYTE	14.11	1.46E-08 ↓	NS
Methanone, dicyclohexyl-	MEDI	16.96	6.10E-07 ↑	NS
*1,3,5-trichloro-Benzene	TCHB	11.86	2.20E-06 ↓	1.56E-04 ↑
*2,5-Cyclohexadiene-1,4-dione, 2,6-bis(1,1-dimethylethyl)-	CHDD	16.23	7.96E-06 ↑	NS
2,2,4-Trimethyl-1,3-pentanediol diisobutyrate	TRPD	18.05	3.64E-05 ↓	NS
2-Hexanone	2-HEX	4.41	1.50E-04 ↓	NS
2,4-Heptanedione	HDIN	8.09	2.51E-04 ↓	NS
2,4-Heptanedione, 6-methyl-	HEDM	9.14	4.62E-04 ↓	NS
*Acetone	ACET	1.58	5.56E-04 ↓	NS
*2-Heptanone	2-HEP	6.4	6.63E-04 ↓	NS
2-Propanol, 2-methyl-	PRPM	1.65	7.59E-04 ↓	NS
2-Butanone	2-BUT	1.99	1.13E-03 ↓	2.45E-02 ↑
8,9-Dehydrothymol	DTHY	12.45	1.15E-03 ↓	NS
1,2-Benzenedicarboxylic acid, bis(2-methylpropyl) ester	BCAE	21.2	1.22E-03 ↓	NS
1,2-Dimethyl-4-oxocyclohex-2-enecarboxaldehyde	DOCR	14.56	1.94E-03 ↑	3.90E-02 ↓
*Dibutyl phthalate	DBPH	22.2	2.99E-03 ↓	5.87E-03 ↑
Benzoic acid	BEAC	11.6	3.03E-03 ↓	NS
10-Dodecanol	DDOL	15.28	3.68E-03 ↓	NS
Propanoic acid, 2-methyl-, 2-ethyl-3-hydroxyhexyl ester	PRAM	14.83	4.30E-03 ↓	NS
2-Pentanone	2-PEN	2.81	4.94E-03 ↓	NS
Prenol	PREN	4.16	7.35E-03 ↓	NS
Decanal	DCAL	12.24	9.86E-03 ↓	NS
4-Undecene, 5-methyl	UDCM	11.54	1.05E-02 ↑	NS
*2-Butanone, 3,3-dimethyl-	2-BDI	3.11	1.10E-02 ↓	4.48E-04 ↓
3-Undecene, 3-methyl-	UNCM	13.23	1.17E-02 ↑	NS
p-Cymene	CYME	9.21	1.30E-02 ↓	NS
Furan, 2-pentyl-	FURP	8.52	1.72E-02 ↓	NS
4-Hexen-3-one, 5-methyl-	4-HEX	7.79	1.79E-02 ↓	NS
2-Hexanone, 5-methyl-	2-HXM	5.74	1.86E-02 ↓	NS
1-Undecanol	UNOL	14.73	1.92E-02 ↓	NS
Undecane, 3-methylene	UNME	11.39	2.07E-02 ↓	NS
*2,4-Di-tert-butylphenol	DTBP	16.79	2.07E-02 ↑	1.30E-04 ↓
p-Mentha-1,5-dien-8-ol	MEOL	11.58	2.16E-02 ↓	NS

2(3H)-Benzofuranone, 3a,4,5,7a-tetrahydro-3,6-dimethyl-	BFTH	13.16	2.22E-02 ↓	NS
Benzene	BENZ	2.57	2.39E-02 ↓	NS
Formamide, N-phenyl-	FORP	12.46	2.56E-02 ↓	NS
o-Toluidine	TOLI	10.14	3.01E-02 ↓	NS
Benzene, 1,4-dimethyl-2-(2-methylpropyl)-	BDMP	13.98	3.19E-02 ↓	NS
Benzoic acid, ethyl ester	BAEE	11.74	3.54E-02 ↓	NS
2,6,6-Trimethyl-2-cyclohexene-1,4-dione	TCDI	11.31	4.23E-02 ↓	3.32E-02 ↑
n-Caproic acid vinyl ester	CAAV	8.36	4.45E-02 ↓	NS
Sesquiterpene	USES	19.6	4.80E-02 ↑	NS
Bisabolol	BISA	19.22	4.91E-02 ↓	NS
Norbornane, 2-isobutyl-	NORB	8.86	4.91E-02 ↓	NS
4-Hydroxy-2,6,6-trimethyl-3-oxocyclohex-1-ene-1-carboxaldehyde	4-HOC	14.39	NS	1.59E-03 ↓
2-Ethyl-5-n-propylphenol	2-EPP	15.95	NS	2.26E-03 ↓
2-Ethyl-4-methylanisole	EMAN	13.44	NS	2.48E-03 ↓
1,4-Benzenediol, 2-methoxy-	BIOM	12.62	NS	3.33E-03 ↓
3,5-di-tert-Butyl-4-hydroxybenzaldehyde	DTBB	20.14	NS	4.12E-03 ↓
2-Undecanone	UNON	13.57	NS	6.14E-03 ↓
3-methoxy-2-(1-methylethyl)-5-(2-methylpropyl) pyrazine	3-MPP	14.78	NS	6.41E-03 ↓
3-Cyclohexene-1-carboxaldehyde	CHCX	9.03	NS	6.76E-03 ↓
3-Thujen-2-ol	3-THU	11.35	NS	8.30E-03 ↓
8,8,9-Trimethyl-deca-3,5-diene-2,7-dione	TDDD	16.07	NS	8.67E-03 ↓
*p-Cymen-8-ol	CYOL	11.96	NS	1.01E-02 ↓
Cyclohexane, 1,2,3-trimethyl-	CHTI	6.81	NS	1.02E-02 ↑
1,3,5-Undecatriene	UNTR	11.89	NS	1.12E-02 ↓
2-n-Heptylcyclopentanone	HCYP	14.35	NS	1.24E-02 ↑
Cyclohexane, 1,2,4-trimethyl-	CTRI	6.13	NS	1.38E-02 ↑
Disulfide, methyl (methylthio)methyl	DSMM	11.06	NS	1.42E-02 ↑
Benzoic acid, 4-ethoxy-, ethyl ester	BZAA	17.02	NS	1.51E-02 ↓
8-Pentadecanone	PNDA	18.99	NS	1.53E-02 ↓
2-Acetyl-5-methylthiophene	AMTH	13.48	NS	1.86E-02 ↓
1(2H)-Naphthalenone, 3-(1,1-dimethylethyl)-3,4-dihydro-	NAPD	18.18	NS	2.11E-02 ↓
2-Nonanone	2-NON	10.46	NS	2.18E-02 ↓
Safranal	SAFR	12.39	NS	2.40E-02 ↓
Cuminal	CUMI	12.76	NS	2.42E-02 ↓
3-Penten-2-ol	PEOL	2.51	NS	2.80E-02 ↑
Propanoic acid, 2-methyl-, 3-hydroxy-2,2,4-trimethylpentyl ester	PRHP	14.53	NS	3.25E-02 ↑
Terpinene	TERP	9.6	NS	3.37E-02 ↑
Hexadecane	HXDA	19.3	NS	3.65E-02 ↑
Ethyl-4-(ethyloxy)-2-oxobut-3-enoate	EOXB	12.82	NS	3.66E-02 ↑
*Thujaplicin	THUJ	13.1	NS	3.74E-02 ↓
Benzene, 1-methoxy-4-(1-methylpropyl)-	BMEP	12.34	NS	4.38E-02 ↓

Supplementary Table S2. Regression analysis results of individual 37 VOCs identified as significantly different (Cancer Week 1 and Week 3), standardized regression coefficients for the same 37 VOCs analyzed by PCR in Figure 5(b) and standardized regression coefficients for the 19 VOCs analyzed by PCR in Figure 5(d).

	Linear Regression of Individual VOCs				PCR of 37 VOCs					PCR of 19 VOCs				
VOC Abbreviation	R2	Adjusted R2	Regression Coeff.	p-value of linear T-stat	Regression Coefficient	Standard Error	T-stat	p-value of linear T-stat	Range CI (95%)	Regression Coefficient	Standard Error	T-stat	p-value	Range CI (95%)
DTBP	0.32	0.3	-2.87	< 0.0001	-0.47	0.11	-4.38	< 0.0001	-0.69 / -0.25	-0.69	0.11	-6.12	< 0.0001	-0.92 / -0.46
AMTH	0.1	0.08	-1.7	0.036	0.16	0.04	4.14	< 0.0001	0.08 / 0.24	0.17	0.04	4.82	< 0.0001	0.10 / 0.24
TDDD	0.13	0.11	-2.03	0.014	0.14	0.03	4.33	< 0.0001	0.07 / 0.20	0.09	0.03	3.39	0.002	0.04 / 0.15
TCHB	0.37	0.35	12.65	< 0.0001	0.06	0.01	5.8	< 0.0001	0.04 / 0.08	0.06	0.01	6.56	< 0.0001	0.04 / 0.08
PEOL	0.03	0	0.8	0.280	0.29	0.09	3.13	0.004	0.10 / 0.48	0.29	0.1	2.98	0.005	0.09 / 0.49
4-HOC	0.24	0.22	-2.6	0.001	-0.12	0.04	-2.92	0.007	-0.21 / -0.04	-0.15	0.04	-3.41	0.002	-0.23 / -0.06
CHCX	0.12	0.1	-1.88	0.021	-0.3	0.11	-2.81	0.010	-0.52 / -0.08	-0.26	0.1	-2.62	0.013	-0.46 / -0.06
CYOL	0.17	0.15	-2.34	0.005	-0.26	0.09	-2.8	0.010	-0.45 / -0.07	-0.32	0.1	-3.07	0.004	-0.53 / -0.11
NAPD	0.07	0.05	-1.57	0.073	0.27	0.1	2.71	0.012	0.07 / 0.48	0.2	0.1	1.93	0.062	-0.01 / 0.41
UNON	0.15	0.13	-2.04	0.007	0.07	0.03	2.46	0.021	0.01 / 0.13	0.12	0.04	3.35	0.002	0.05 / 0.19
SAFR	0.08	0.06	-1.83	0.061	0.21	0.09	2.41	0.024	0.03 / 0.39	0.06	0.08	0.72	0.476	-0.10 / 0.21
BIOM	0.15	0.13	-2.1	0.008	0.11	0.05	2.32	0.029	0.01 / 0.20	0.16	0.05	3.41	0.002	0.06 / 0.25
2-BDI	0.22	0.2	-2.8	0.001	-0.16	0.07	-2.25	0.033	-0.30 / -0.01	0.04	0.05	0.71	0.481	-0.07 / 0.15
HXDA	0.09	0.07	1.74	0.047	0.17	0.07	2.25	0.033	0.01 / 0.32	0.1	0.08	1.23	0.228	-0.07 / 0.27
THUJ	0.06	0.04	-1.81	0.103	-0.18	0.08	-2.22	0.036	-0.35 / -0.01	-0.1	0.08	-1.26	0.215	-0.25 / 0.06
HCYP	0.11	0.09	1.73	0.026	0.22	0.1	2.14	0.043	0.01 / 0.43	0.28	0.1	2.78	0.009	0.07 / 0.48
TERP	0.02	0	0.68	0.334	0.21	0.1	2.1	0.046	0.00 / 0.41	0.18	0.09	2.01	0.053	0.00 / 0.36
EMAN	0.18	0.16	-2.43	0.004	-0.24	0.12	-2.08	0.048	-0.48 / 0.00	-0.26	0.11	-2.42	0.021	-0.47 / -0.04
2-NON	0.08	0.05	-1.9	0.067	0.17	0.08	2.08	0.048	0.00 / 0.34	0.19	0.08	2.32	0.026	0.02 / 0.36
DTBB	0.16	0.14	-2.21	0.007	-0.13	0.06	-2.01	0.055	-0.26 / 0.00					
DBPH	0.22	0.2	2.89	0.001	0.16	0.08	2.02	0.055	0.00 / 0.32					
CUMI	0.09	0.06	-1.48	0.052	0.29	0.15	1.94	0.064	-0.02 / 0.59					
PNDA	0.11	0.09	-2.59	0.028	0.12	0.06	1.91	0.068	-0.01 / 0.25					
DSMM	0.05	0.03	0.91	0.132	0.16	0.1	1.64	0.113	-0.04 / 0.36					
CTRI	0.08	0.06	2.13	0.054	0.09	0.06	1.61	0.121	-0.03 / 0.20					
EOXB	0	-0.02	0.24	0.766	-0.14	0.09	-1.51	0.144	-0.32 / 0.05					
CHTI	0.08	0.06	2.01	0.052	0.09	0.06	1.5	0.145	-0.03 / 0.20					
DOCR	0.04	0.02	-1.19	0.193	0.15	0.1	1.41	0.170	-0.07 / 0.36					
2-EPP	0.16	0.14	-2.19	0.006	-0.08	0.07	-1.24	0.225	-0.21 / 0.05					
BMEP	0.12	0.1	-1.82	0.020	-0.1	0.09	-1.03	0.312	-0.29 / 0.10					
3-MPP	0.15	0.13	-2.15	0.0079	-0.04	0.07	-0.58	0.565	-0.17 / 0.10					
3-THU	0.16	0.14	-2.34	0.006	-0.04	0.09	-0.51	0.617	-0.22 / 0.14					
PRAM	0.2	0.19	2.9	0.001	0.03	0.07	0.42	0.681	-0.11 / 0.17					
2-BUT	0.07	0.05	1.52	0.082	0.03	0.09	0.39	0.698	-0.15 / 0.21					
UNTR	0.11	0.08	-1.58	0.030	-0.03	0.08	-0.37	0.718	-0.20 / 0.14					
TCDI	0.07	0.05	1.52	0.074	-0.02	0.09	-0.24	0.814	-0.20 / 0.16					
BZAA	0.1	0.08	-1.66	0.036	0	0.05	-0.08	0.936	-0.12 / 0.11					

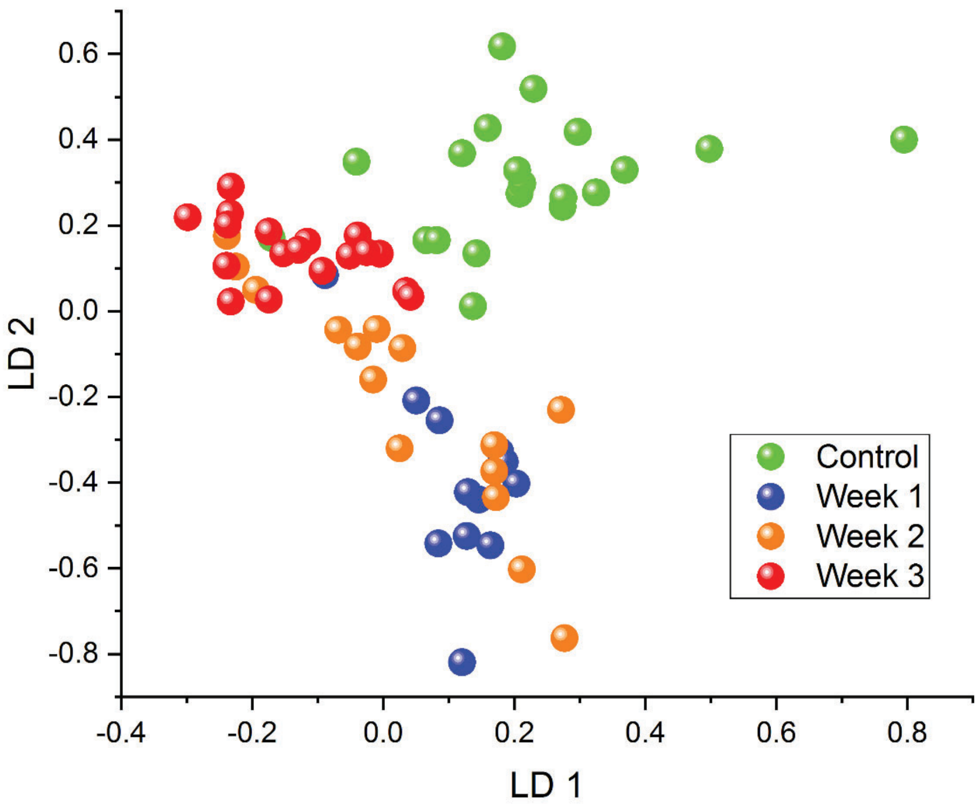

Figure S1: 2D LDA plot with Cancer Week 2 samples tested utilizing the LDA model built to discriminate Control, Cancer Week 1, and Cancer Week 3.
